# Factors associated with the nutritional status of children under 5 years of age in Guinea between 2005 and 2018

**DOI:** 10.1017/S1368980022002622

**Published:** 2023-03

**Authors:** Salifou Talassone Bangoura, Muriel Rabilloud, Alioune Camara, Séphora Campoy, Mamoudou Condé, Philippe Vanhems, Kadio Jean-Jacques Olivier Kadio, Abdoulaye Touré, Nagham Khanafer

**Affiliations:** 1 Centre de Recherche et de Formation en Infectiologie de Guinée (CERFIG), Campus Universitaire Hadja Mafory Bangoura, Donka, Conakry, BP: 6629P, Guinée; 2 Chaire de Santé Publique, Université Gamal Abdel Nasser de Conakry, Conakry, Guinée; 3 Département des Sciences Pharmaceutiques et Biologiques, Université Gamal Abdel Nasser de Conakry, Conakry, Guinée; 4 Université Lyon 1, CNRS, UMR5558, Laboratoire de Biométrie et Biologie Évolutive, Équipe Biostatistique-Santé, Villeurbanne, France; 5 Hospices Civils de Lyon, Pôle Santé Publique, Service de Biostatistique et Bioinformatique, Lyon, France; 6 Santé publique, Epidémiologie et Ecologie Evolutive des Maladies Infectieuses, Centre International de Recherche en Infectiologie (CIRI), INSERM U1111-UCBL 1-ENS, Lyon, France; 7 Department of Hygiene, Epidemiology, and Prevention, Edouard Herriot Hospital, Hospices Civils de Lyon, Lyon, France

**Keywords:** Children, Nutritional status, Factors associated, Guinea

## Abstract

**Objective:**

To determine the factors associated with the nutritional status of children under 5 years of age in Guinea between 2005 and 2018.

**Design:**

Data from the 2005, 2012 and 2018 Guinea Demographic and Health Surveys (DHS) were used for this study. Three anthropometric indicators (stunting, underweight and wasting) were assessed according to the 2006 WHO Child Growth Standards and analysed according to the year, the characteristics of the household, the child and the mother using multivariate logistic regression.

**Setting:**

Data were collected in the capital Conakry and in the seven administrative regions of Guinea.

**Participants:**

The study included children under 5 years of age for whom height and weight were available: 2765 (DHS-2005), 3220 (DHS-2012) and 3551 (DHS-2018).

**Results:**

Analysis of the data from the three surveys showed that children living in rural areas were more likely to be stunted than children living in urban areas (OR = 1·32, 95 % CI (1·08, 1·62)). Similarly, the children from middle, poor and the poorest households were more likely to be stunted and underweight than children from richest households. The chance to stunting increased with age in the first 3 years. However, the chance to wasting decreased with age. Children in all age groups were more likely of being underweight. Children of thin mothers were more likely to be both wasted (OR = 2·0, 95 % CI (1·5, 2·6)) and underweight (OR = 1·9, 95 % CI (1·5, 2·3)).

**Conclusion:**

The implementation of targeted interventions adapted to the observed disparities could considerably improve the nutritional status of children and mothers.

The early years of life, from birth to 5 years of age, are critical for the development of physical, mental and emotional characteristics into adulthood^(1,2)^. Malnutrition during this period is one of the most important global public health problems^(3)^; it is detrimental to proper brain development and linear growth of children and has both short- and long-term consequences for their health but also the economic productivity of countries^(4)^. About 45 % of deaths before the age of 5 years are related to malnutrition, and the majority of these occur mainly in low- and middle-income countries, particularly in Africa and South Asia^(5)^. International efforts such as the UN sustainable development goals are focusing attention and resources on this important issue^(6)^; the sustainable development goals 2·2 focuses on improving nutrition and eliminating all forms of malnutrition, including achieving by 2025 internationally agreed targets on stunting and wasting in children under 5 years of age and meeting the nutritional needs of adolescent girls, pregnant and lactating women, and older people^(7)^. However, progress is insufficient to meet global targets; for instance, the latest Global Nutrition Report indicated that 21·3 % of children under 5 years of age were stunted and 6·9 % wasted; in sub-Saharan Africa, it is estimated that 31·1 % of children under 5 years of age were stunted and 6·3 % were wasted^(8)^. In Guinea, the various forms of malnutrition affect every year thousands of children under the age of 5 years with more than 204 000 children affected by severe acute malnutrition and about 750 000 children by chronic malnutrition and are associated with more than half of all child deaths in the country^(9)^. Despite this alarming situation, the coverage of essential curative, preventive and promotional nutrition interventions remains low^(9)^. This was compounded by the Ebola virus disease epidemic that, between March 2014 and April 2016, contributed to the deterioration of the nutritional status of children and the weakening of the health system. Faced with the nutritional variations to which children have been exposed, no study has analysed the factors associated with the nutritional status of children under the age of 5 years post-Ebola and the temporal variations of this effect on these factors. The objective of this study was to determine the factors associated with stunting, underweight and wasting according to household, child and mother characteristics.

## Methods

### Study design and population

This study used data from the last three Demographic and Health Surveys (DHS) conducted in Guinea in 2005, 2012 and 2018 by the National Statistics Institute of Guinea (*Institut National de la Statistique*, INS) with technical assistance from the DHS programme (Program DHS, ICF/USAID). These surveys used two-stage stratified cluster sampling methods (region and place of residence). The weight and height of the children were measured and collected during these repeated cross-sectional studies. Weight was measured using electronic scales (Seca, Hamburg, Germany), while height measurements were taken using graduated height scales. Children under 2 years of age were measured lying down, while older children were measured standing up^(10–12)^. Data of children under 5 years of age who had a weight and height measurement available were analysed.

### Study sampling

A two-stage stratified random sampling design was used for the 2005, 2012 and 2018 DHS-Guinea. At the first stage, clusters or areas of enumeration were drawn in each stratum from the list established during the mapping work for the second and third General census of population and habitation in Guinea (Récensement Général de la Population et de l’Habitation) in 2004 and 2014, respectively. At the second stage, households were drawn in clusters in each stratum. Within the selected households, women aged 15–49 years were interviewed (7954, 9142 and 10 874 in 2005, 2012 and 2018, respectively) and children under 5 years were registered (6364, 7039 and 7951 in 2005, 2012 and 2018, respectively). However, measurements of weight (in kilograms) and height (in centimetres) were taken for 2765 children under 5 years of age (DHS-2005), 3220 (DHS-2012) and 3551 (DHS-2018)^(10–12)^.

### Outcome criteria

The nutritional status of the children was assessed, as recommended by the WHO, by three anthropometric indices: height-for-age, weight-for-height and weight-for-age. A child was considered stunted, wasted or underweight when, respectively, the value of the height-for-age-score, the weight-for-height-score or the weight-for-age-score was less than −2 sd below the median of the reference population as defined by the WHO in 2006^(13)^.

### Studied factors

#### The year of the anthropometric measurements

2005, 2012 and 2018.

#### The household characteristics

The region (Boké, Conakry, Faranah, Kankan, Kindia, Labé, Mamou and N’Zérékoré), the place of residence and the household wealth grouped into five categories according to the quintiles of the wealth score used by the DHS wealth index: poorest, poor, middle, rich and richest^(14)^. The latter was built using the following information: household ownership of certain goods (television, radio, car, etc.) and certain housing characteristics (availability of electricity, type of drinking water supply, type of toilet, flooring material, number of rooms used for sleeping, type of fuel for cooking, etc.). A score was assigned to each characteristic, and the overall score for each household was obtained by adding the scores^(14)^.

#### Characteristics of children

Age was categorised as follows: <6 months, 6–11 months, 12–23 months, 24–35 months, 36–47 months and 48–59 months; sex; clinical signs such as diarrhoea, fever and cough in last 2 weeks.

#### Characteristics of the mother

Education level was categorised into three levels: unschooled, primary level (women who had stopped schooling between first and sixth grade) and secondary level or above, and BMI was calculated by dividing the weight in kilograms (kg) by the square of the height in metres (m) and categorised according to the WHO classification standards^(15)^: underweight (BMI < 18·5 kg/m^2^), normal weight (BMI between 18·5 and 24·9 kg/m²) and overweight (BMI ≥ 25 kg/m²).

### Statistical analysis

The data were analysed using the statistical software R version 1.4.1106R: A language and environment for statistical computing, and R Foundation for Statistical Computing (URL https://www.R-project.org/). Descriptive analyses of household, child and maternal characteristics were performed separately for each survey. Height-for-age, weight-for-height and weight-for-age *Z*-scores were calculated for children who had available height and weight measurements, and the height-for-age < -6 or > 6, weight-for-height < -5 or > 5 and weight-for-age < -6 or > 5 were excluded according to the WHO standards^(13)^. The exclusion was done by *Z*-score and not by child. Univariate and multivariate logistic regression models were used to identify factors associated with stunting, wasting and underweight. The variables with a *P*-value ≤ 20 % in the univariate analysis or considered clinically important were retained for the multivariate analysis by stepwise ascending method. The interactions between the variables retained in the multivariate analysis were tested. The different models were then compared using the likelihood ratio test to select the goodness of fit of the model. The effects of the studied factors were quantified by OR and their 95 % CI. The sample design of the three DHS was taken into account in the logistic regression models to allow the extrapolation of the results to the whole Guinean population.

### Ethical considerations

The protocol and questionnaires of the DHS-2005, 2012 and 2018 have been approved by the National Ethics Committee and endorsed by the ICF Ethics Committee (International Review Board), and the informed and voluntary consent of the parents was obtained for all children. The data were obtained, following a request on the DHS website (URL: https://www.dhsprogram.com/data/available-datasets.cfm). However, no formal ethical approval was obtained as the study involved secondary analysis of publicly available data.

## Results

**Fig. 1:**
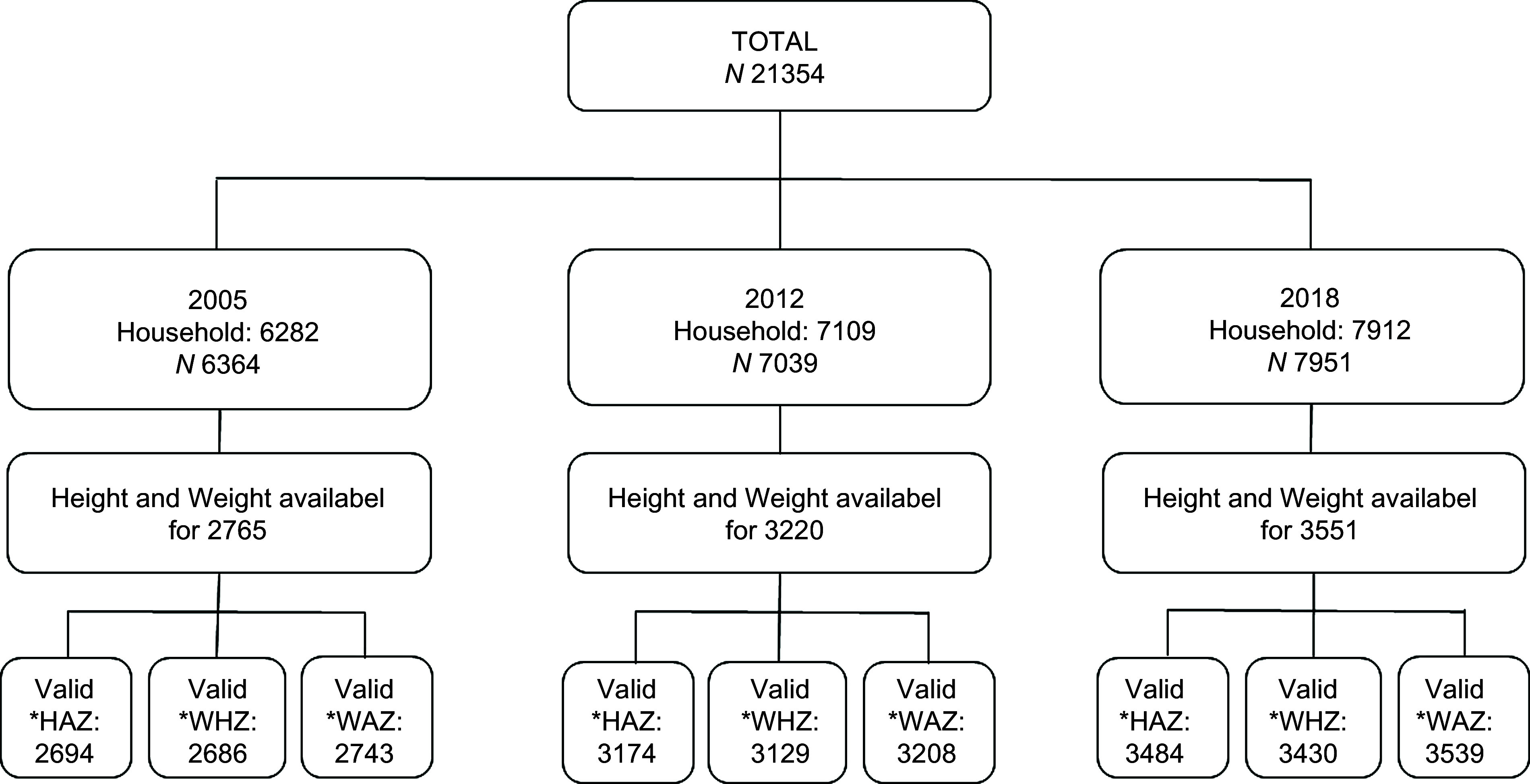
Data selection flowchart for children under 5 years of age included in the DHS of 2005, 2012 and 2018, Guinea. *HAZ (*Z*-score height-for-age); WHZ (*Z*-score weight-for-height); WAZ (*Z*-score weight-for-age)

A total of 21 354 children under 5 years of age were included in the three DHS. Height and weight measurements were available for 2765 children in 2005, 3220 in 2012 and 3551 in 2018. Valid height-for-age *Z*-scores were obtained for 2694 (97·4 %) children in 2005, 3174 (98·6 %) in 2012 and 3484 (98·1 %) in 2018. Similarly, valid weight-for-height *Z*-scores were obtained for 2686 (97·1 %) children in 2005, 3129 (97·2 %) in 2012 and 3430 (96·6 %) in 2018. Valid weight-for-age *Z*-scores were obtained for 2743 (99·2 %) children in 2005, 3208 (99·6 %) in 2012 and 3539 (99·7 %) in 2018 (Fig. 1).

During the three DHS, more than 70 % of children under 5 years old were included in rural areas. About 17 % of the children lived in the Kankan region and 24 % of the children were from poorest households. The majority of the children were boys (51·4 %) and between 12 and 23 months of age (20·7 %). Over 80 % of the children’s mothers were unschooled, and only 6·4 % of mothers were thin (Table 1).

**Table 1:** Description of household, child and maternal characteristics according to the DHS 2005, 2012 and 2018 Guinea

Characteristics	2005 (*n* 2765)		2012 (*n* 3220)		2018 (*n* 3551)		Overall (*n* 9536)	
	*n*	%	*n*	%	*n*	%	*n*	%
Household characteristics								
Place of residence								
Urban	610	22·1	902	28·0	1005	28·3	2517	26·4
Rural	2155	77·9	2318	72·0	2546	71·7	7019	73·6
Region								
Boké	338	12·2	339	10·5	508	14·3	1185	12·4
Conakry	211	7·6	306	9·5	304	8·6	821	8·6
Faranah	383	13·9	471	14·6	495	13·9	1349	14·1
Kankan	492	17·8	578	18·0	542	15·3	1612	16·9
Kindia	398	14·4	391	12·1	418	11·8	1207	12·7
Labé	261	9·4	369	11·5	461	13·0	1091	11·4
Mamou	275	9·9	347	10·8	397	11·2	1019	10·7
N’zérékoré	407	14·7	419	13·0	426	12·0	1252	13·1
Household wealth								
Richest	378	13·7	411	12·8	523	14·7	1312	13·8
Rich	517	18·7	733	22·8	661	18·6	1911	20·0
Middle	603	21·8	627	19·5	684	19·3	1914	20·1
Poor	598	21·6	676	21·0	803	22·6	2077	21·8
Poorest	669	24·2	773	24·0	880	24·8	2322	24·3
Child characteristics								
Age (months)								
<6	397	14·3	419	13·0	452	12·7	1267	13·3
6–11	304	11·0	351	10·9	327	9·2	982	10·3
12–23	572	20·7	673	20·9	733	20·6	1978	20·7
24–35	533	19·3	589	18·3	614	17·3	1736	18·2
36–47	461	16·7	594	18·4	746	21·0	1801	18·9
48–59	498	18·0	594	18·4	679	19·1	1771	18·6
Sex								
Female	1369	49·5	1531	47·5	1735	48·9	4635	48·6
Male	1396	50·5	1689	52·5	1816	51·1	4901	51·4
Diarrhoea in last 2 weeks								
No	2331	84·3	2657	82·5	3130	88·1	8118	85·1
Yes	418	15·1	545	16·9	417	11·7	1380	14·5
Missing	16	0·6	18	0·6	4	0·1	38	0·4
Fever in last 2 weeks								
No	1832	66·3	2156	67·0	2977	83·8	6965	73·0
Yes	918	33·2	1047	32·5	571	16·1	2536	26·6
Missing	15	0·5	17	0·5	3	0·1	35	0·4
Cough in last 2 weeks								
No	2123	76·8	2544	79·0	3143	88·5	7810	81·9
Yes	629	22·7	655	20·3	406	11·4	1690	17·7
Missing	13	0·5	21	0·7	2	0·1	36	0·4
Mother characteristics								
Education								
Secondary or above	120	4·3	307	9·5	424	11·9	851	8·9
Primary	210	7·6	365	11·3	390	11·0	965	10·1
Unschooled	2435	88·1	2548	79·1	2737	77·1	7720	81·0
BMI (kg/m²)								
Thin (<18·5)	203	7·3	223	6·9	189	5·3	615	6·4
Normal (18·5–24·9)	2128	77·0	2310	71·7	2343	66·0	6781	71·1
Overweight (≥25)	283	10·2	542	16·8	889	25·0	1714	18·0
Missing	151	5·5	145	4·5	130	3·7	426	4·5

Compared with the 2005 and 2012 surveys, children from middle (OR = 1·8, 95 % CI (1·1, 2·8)), poor (OR = 1·7, 95 % CI (1·1, 2·6)) and poorest (OR = 2·0, 95 % CI (1·3, 3·2)) households were more likely to be stunted in 2018 than children from richest households. In all surveys, the children’s likelihood of stunting increased significantly with age in the first 36 months, with higher likelihood in 2005 and 2012. Boys were more likely than girls to be stunted in the 2005 (OR = 1·3, 95 % CI (1·1, 1·5)) and 2018 (OR = 1·4, 95 % CI (1·2, 1·7)) surveys. Children of thin mothers were more likely to be stunted in 2012 (OR = 1·6, 95 % CI (1·1, 2·4)) (Table 2).

**Table 2:** Multivariate logistic regression of factors associated with stunting, in children under 5 years of age included in DHS of 2005, 2012 and 2018, Guinea

Stunting (height-for-age <-2 sd)						
Characteristics	2005		2012		2018	
	Adjusted OR	95 % CI	Adjusted OR	95 % CI	Adjusted OR	95 % CI
Household characteristics						
Place of residence						
Urban	1·0		1·0		1·0	
Rural	1·3	0·9, 1·9	1·4	1·0, 2·0	1·1	0·8, 1·6
Region						
Conakry	1·0		1·0		1·0	
Boké	0·6	0·3, 1·1	1·0	0·5, 1·9	1·2	0·7, 2·0
Faranah	0·7	0·4, 1·4	1·2	0·6, 2·3	0·8	0·5, 1·2
Kankan	1·3	0·7, 2·6	1·3	0·7, 2·5	0·7	0·5, 1·2
Kindia	0·9	0·5, 1·7	1·2	0·6, 2·2	0·7	0·5, 1·2
Labé	0·9	0·5, 1·8	1·3	0·6, 2·6	0·8	0·5, 1·2
Mamou	0·8	0·4, 1·6	1·9	1·0, 3·7	0·7	0·4, 1·2
N’zérékoré	1·3	0·7, 2·4	1·8	1·0, 3·3	1·0	0·6, 1·7
Household wealth						
Richest	1·0		1·0		1·0	
Rich	1·2	0·7, 2·1	1·1	0·6, 2·1	1·3	0·9, 1·9
Middle	1·6	0·9, 2·7	1·2	0·6, 2·4	1·8	1·1, 2·8
Poor	1·8	1·0, 3·2	1·8	0·9, 3·7	1·7	1·1, 2·6
Poorest	1·9	1·0, 3·4	1·5	0·8, 3·0	2·0	1·3, 3·2
Child characteristics						
Age (months)						
<6	1·0		1·0		1·0	
6–11	1·6	1·1, 2·4	1·6	0·9, 2·7	1·2	0·8, 1·8
12–23	5·8	3·9, 8·8	3·2	2·1, 4·9	2·3	1·7, 3·3
24–35	9·2	6·1, 13·7	6·0	4·0, 9·1	2·4	1·7, 3·4
36–47	6·8	4·5, 10·2	6·0	4·1, 9·0	2·2	1·5, 3·1
48–59	6·8	4·6, 10·1	4·8	3·2, 7·2	2·2	1·5, 3·1
Sex						
Female	1·0		1·0		1·0	
Male	1·3	1·1, 1·5	1·2	1·0, 1·5	1·4	1·2, 1·7
Diarrhoea in last 2 weeks						
No	1·0		1·0		1·0	
Yes	1·1	0·9, 1·4	1·1	0·8, 1·5	1·3	1·0, 1·7
Fever in last 2 weeks						
No	1·0		1·0		1·0	
Yes	1·1	0·9, 1·4	1·0	0·8, 1·3	1·2	0·9, 1·5
Cough in last 2 weeks						
No	1·0		1·0		1·0	
Yes	0·9	0·7, 1·2	1·0	0·7, 1·3	1·0	0·7, 1·4
Mother characteristics						
Education						
Secondary or above	1·0		1·0		1·0	
Primary	1·2	0·6, 2·2	1·2	0·7, 1·9	1·3	0·9, 1·9
Unschooled	1·3	0·8, 2·3	1·3	0·8, 1·9	1·3	1·0, 1·8
BMI (kg/m²)						
Normal (18·5–24·9)	1·0		1·0		1·0	
Thin (<18·5)	1·4	1·0, 2·1	1·6	1·1, 2·4	0·9	0·6, 1·2
Overweight (≥25)	0·8	0·6, 1·0	0·9	0·7, 1·2	0·9	0·7, 1·1

Compared with children aged less than 6 months, children aged 6–11 months had a higher chance of being wasted in 2005 (OR = 1·7, 95 % CI (1·1, 2·6)), but this changed in 2018 (OR = 0·6, 95 % CI (0·4, 1·0)). In all surveys, children aged between 36 and 59 months were significantly less likely to be wasted. However, the children who had had fever in the last 2 weeks preceding the surveys had more chance of being wasted than those did not have fever, and this was significant in 2018. Children of thin mothers were more likely to be wasted than children of normal-weight mothers; this difference was significant in 2005 (OR = 2·3, 95 % CI (1·4, 3·6)) and 2018 (OR = 2·7, 95 % CI (1·7, 4·1)) surveys (Table 3).

**Table 3:** Multivariate logistic regression of factors associated with wasting in children under 5 years of age included in DHS of 2005, 2012 and 2018, Guinea

Wasting (weight-for-height <-2 sd)						
Characteristics	2005		2012		2018	
	Adjusted OR	95 % CI	Adjusted OR	95 % CI	Adjusted OR	95 % CI
Household characteristics						
Place of residence						
Urban	1·0		1·0		1·0	
Rural	1·0	0·6, 1·6	1·3	0·8, 2·1	0·8	0·5, 1·4
Region						
Conakry	1·0		1·0		1·0	
Boké	0·4	0·1, 1·0	0·9	0·4, 1·9	0·5	0·2, 1·1
Faranah	1·0	0·4, 2·4	1·0	0·4, 2·2	0·4	0·2, 0·9
Kankan	0·9	0·3, 2·4	2·2	1·0, 4·7	0·8	0·4, 1·7
Kindia	0·6	0·2, 1·3	0·7	0·3, 1·5	0·5	0·3, 1·1
Labé	0·7	0·3, 1·8	1·1	0·5, 2·5	0·4	0·2, 0·9
Mamou	0·2	0·1, 0·7	1·0	0·4, 2·2	0·5	0·2, 1·0
N’zérékoré	0·6	0·3, 1·6	0·8	0·3, 1·7	0·6	0·3, 1·3
Household wealth						
Richest	1·0		1·0		1·0	
Rich	1·3	0·5, 3·2	1·1	0·5, 2·2	1·4	0·8, 2·5
Middle	1·2	0·5, 3·1	1·0	0·5, 2·1	1·4	0·7, 2·8
Poor	1·2	0·5, 3·0	1·1	0·5, 2·4	1·6	0·8, 3·3
Poorest	1·5	0·6, 3·9	1·0	0·5, 2·3	1·3	0·6, 2·8
Child characteristics						
Age (months)						
<6	1·0		1·0		1·0	
6–11	1·7	1·1, 2·6	1·2	0·7, 1·9	0·6	0·4, 1·0
12–23	1·0	0·6, 1·5	1·5	1·0, 2·4	0·6	0·4, 0·8
24–35	0·7	0·4, 1·1	0·7	0·4, 1·2	0·6	0·4, 0·9
36–47	0·5	0·3, 0·8	0·3	0·2, 0·6	0·4	0·2, 0·5
48–59	0·2	0·1, 0·4	0·3	0·2, 0·6	0·4	0·3, 0·7
Sex						
Female	1·0		1·0		1·0	
Male	1·3	1·0, 1·6	1·2	0·9, 1·5	1·1	0·8, 1·4
Diarrhoea in last 2 weeks						
No	1·0		1·0		1·0	
Yes	0·8	0·5, 1·1	1·2	0·8, 1·8	1·1	0·8, 1·5
Fever in last 2 weeks						
No	1·0		1·0		1·0	
Yes	1·1	0·7, 1·5	0·9	0·7, 1·3	1·6	1·2, 2·1
Cough in last 2 weeks						
No	1·0		1·0		1·0	
Yes	1·4	0·9, 2·0	1·1	0·8, 1·4	1·0	0·7, 1·4
Mother characteristics						
Education						
Secondary or above	1·0		1·0		1·0	
Primary	1·0	0·4, 2·5	1·2	0·7, 2·1	0·9	0·5, 1·6
Unschooled	0·9	0·4, 1·9	1·0	0·5, 1·7	1·4	1·0, 2·1
BMI (kg/m²)						
Normal (18·5–24·9)	1·0		1·0		1·0	
Thin (<18·5)	2·3	1·4, 3·6	1·4	0·9, 2·2	2·7	1·7, 4·1
Overweight (≥25)	0·9	0·6, 1·5	0·7	0·5, 1·1	0·9	0·6, 1·3

Children from non-wealthy households (middle, poor and poorest) were more likely to be underweight than children from richest households; this difference was significant in 2012 and 2018. Compared with children under 6 months of age, all children aged 6 months and over were likely to be underweight; this difference was NS in 2012 for children aged 6–11 months and in 2018 for those aged 36–47 months. The likelihood of being underweight was significantly higher for children who had had diarrhoea in the last 2 weeks preceding the surveys compared with those who did not have diarrhoea in 2012 (OR = 1·4, 95 % CI (1·1, 1·9)) and in 2018 (OR = 1·4, 95 % CI (1·1, 1·9)). Similarly, children who had had fever in the last 2 weeks preceding the surveys had more chance of being underweight than those who did not have fever; this difference was significant in 2018. In all three surveys, children of thin mothers were more likely to be underweight than those of normal-weight mothers (Table 4).

**Table 4:** Multivariate logistic regression of factors associated with underweight in children under 5 years of age included in DHS of 2005, 2012 and 2018, Guinea

Underweight (weight-for-age <-2 sd)						
Characteristics	2005		2012		2018	
	Adjusted OR	95 % CI	Adjusted OR	95 % CI	Adjusted OR	95 % CI
Household characteristics						
Place of residence						
Urban	1·0		1·0		1·0	
Rural	1·5	1·0, 2·4	1·2	0·7, 2·0	0·9	0·6, 1·3
Region						
Conakry	1·0		1·0		1·0	
Boké	0·4	0·2, 0·8	0·4	0·2, 0·9	1·0	0·6, 1·7
Faranah	0·8	0·4, 1·6	0·5	0·2, 1·1	0·7	0·4, 1·3
Kankan	0·9	0·5, 1·9	1·0	0·5, 2·0	1·0	0·6, 1·7
Kindia	0·6	0·3, 1·2	0·5	0·2, 1·2	0·6	0·3, 1·1
Labé	0·6	0·3, 1·2	0·6	0·3, 1·3	0·7	0·4, 1·3
Mamou	0·4	0·2, 0·9	0·6	0·3, 1·4	0·8	0·5, 1·4
N’zérékoré	0·9	0·4, 1·7	0·6	0·3, 1·3	0·5	0·3, 0·8
Household wealth						
Richest	1·0		1·0		1·0	
Rich	1·0	0·5, 1·9	3·2	1·5, 6·6	1·6	1·0, 2·6
Middle	1·0	0·6, 2·0	3·1	1·4, 7·2	2·1	1·2, 3·8
Poor	1·3	0·7, 2·6	4·7	2·0, 10·8	2·0	1·1, 3·7
Poorest	1·4	0·7, 2·7	3·7	1·6, 8·5	2·1	1·1, 3·8
Child characteristics						
Age (months)						
<6	1·0		1·0		1·0	
6–11	1·9	1·1, 3·1	1·5	0·8, 2·6	1·7	1·1, 2·7
12–23	2·4	1·6, 3·6	3·0	1·9, 4·5	2·0	1·4, 3·0
24–35	3·1	2·0, 4·7	2·6	1·7, 3·9	2·1	1·4, 3·1
36–47	2·4	1·6, 3·6	2·5	1·6, 3·9	1·4	0·9, 2·0
48–59	2·5	1·6, 3·8	2·7	1·7, 4·3	1·7	1·2, 2·5
Sex						
Female	1·0		1·0		1·0	
Male	1·3	1·0, 1·6	1·0	0·8, 1·3	1·2	1·0, 1·5
Diarrhoea in last 2 weeks						
No	1·0		1·0		1·0	
Yes	1·2	0·9, 1·6	1·4	1·1, 1·9	1·4	1·1, 1·9
Fever in last 2 weeks						
No	1·0		1·0		1·0	
Yes	1·3	1·0, 1·7	1·0	0·8, 1·2	1·4	1·1, 1·8
Cough in last 2 weeks						
No	1·0		1·0		1·0	
Yes	1·2	0·8, 1·5	1·1	0·9, 1·5	0·9	0·7, 1·3
Mother characteristics						
Education						
Secondary or above	1·0		1·0		1·0	
Primary	1·0	0·5, 2·3	1·3	0·7, 2·5	1·3	0·8, 2·1
Unschooled	1·2	0·6, 2·4	1·3	0·7, 2·5	1·5	1·0, 2·3
BMI (kg/m²)						
Normal (18·5–24·9)	1·0		1·0		1·0	
Thin (<18·5)	2·3	1·6, 3·4	1·7	1·2, 2·4	1·7	1·1, 2·5
Overweight (≥25)	0·6	0·4, 1·0	0·7	0·5, 1·0	0·6	0·5, 0·8

Multivariate analysis of all data from the three surveys showed that children were significantly less likely to be stunted or underweight in 2012 and 2018 than in 2005. Children living in rural areas were more likely to be stunted than children living in urban areas (OR = 1·3, 95 % CI (1·1, 1·6)). Children from middle (OR = 1·5, 95 % CI (1·1, 2·0)), poor (OR = 1·7, 95 % CI (1·3, 2·4)) and the poorest (OR = 1·7, 95 % CI (1·3, 2·4)) households were more likely to be stunted than children from richest households. Similarly, the children from rich (OR = 1·6, 95 % CI (1·2, 2·3)) middle (OR = 1·7, 95 % CI (1·2, 2·5)), poor (OR = 2·1, 95 % CI (1·4, 3·0)) and poorest (OR = 2·0, 95 % CI (1·4, 3·0)) households were more likely to be underweight than children from the richest households. The likelihood of being stunted increased with age in the first 3 years of life. This difference changed very little for the older age groups. However, the likelihood of being wasted decreased with age. Children in all age groups were likely to be underweight. This likelihood was significantly higher for children aged 12–23 and 24–35 months. Boys were more likely than girls to be stunted (OR = 1·3, 95 % CI (1·2, 1·4)). Compared with their reference categories, children who had had diarrhoea and fever in the last 2 weeks preceding the surveys had more chance of being underweight. Similarly, children of thin mothers were more likely to be both wasted and underweight compared with children of normal-weight mothers (Table 5).

**Table 5:** Multivariate logistic regression of factors associated with stunting, wasting and underweight in children under 5 years of age included in DHS of 2005, 2012 and 2018, Guinea: grouped data

Characteristics	Stunting (height-for-age <-2 sd)		Wasting (weight-for-height <-2 sd)		Underweight (weight-for-age <-2 sd)	
	Adjusted OR	95 % CI	Adjusted OR	95 % CI	Adjusted OR	95 % CI
Year of survey						
2005	1·0		1·0		1·0	
2012	0·6	0·6, 0·7	0·9	0·8, 1·2	0·7	0·6, 0·9
2018	0·7	0·6, 0·8	1·0	0·8, 1·2	0·7	0·6, 0·9
Household characteristics						
Place of residence						
Urban	1·0		1·0		1·0	
Rural	1·3	1·1, 1·6	1·0	0·8, 1·3	1·2	0·9, 1·5
Region						
Conakry	1·0		1·0		1·0	
Boké	0·9	0·7, 1·3	0·5	0·3, 0·9	0·6	0·4, 0·9
Faranah	0·9	0·6, 1·2	0·7	0·4, 1·1	0·7	0·5, 1·0
Kankan	1·1	0·8, 1·5	1·2	0·7, 1·8	1·0	0·7, 1·5
Kindia	0·9	0·7, 1·3	0·6	0·4, 0·9	0·6	0·4, 0·9
Labé	0·9	0·7, 1·3	0·6	0·4, 1·0	0·7	0·4, 1·0
Mamou	1·0	0·7, 1·5	0·5	0·3, 0·8	0·7	0·4, 1·0
N’zérékoré	1·3	0·9, 1·8	0·6	0·4, 1·0	0·7	0·5, 1·0
Household wealth						
Richest	1·0		1·0		1·0	
Rich	1·2	0·9, 1·6	1·3	0·9, 2·0	1·6	1·2, 2·3
Middle	1·5	1·1, 2·0	1·3	0·8, 2·0	1·7	1·2, 2·5
Poor	1·7	1·3, 2·4	1·3	0·8, 2·1	2·1	1·4, 3·0
Poorest	1·7	1·3, 2·4	1·4	0·9, 2·2	2·0	1·4, 3·0
Child characteristics						
Age (months)						
<6	1·0		1·0		1·0	
6–11	1·4	1·1, 1·8	1·1	0·8, 1·4	1·6	1·2, 2·2
12–23	3·4	2·7, 4·3	0·9	0·7, 1·2	2·4	1·9, 3·0
24–35	4·8	3·8, 6·0	0·7	0·5, 0·9	2·4	1·9, 3·1
36–47	4·2	3·3, 5·2	0·4	0·3, 0·5	1·9	1·5, 2·4
48–59	3·9	3·2, 4·9	0·4	0·3, 0·5	2·2	1·7, 2·8
Sex						
Female	1·0		1·0		1·0	
Male	1·3	1·2, 1·4	1·2	1·0, 1·3	1·2	1·0, 1·3
Diarrhoea in last 2 weeks						
No	1·0		1·0		1·0	
Yes	1·2	1·0, 1·4	1·0	0·8, 1·3	1·3	1·1, 1·6
Child characteristics						
Fever in last 2 weeks						
No	1·0		1·0		1·0	
Yes	1·1	0·9, 1·2	1·2	1·0, 1·4	1·2	1·1, 1·4
Cough in last 2 weeks						
No	1·0		1·0		1·0	
Yes	0·9	0·8, 1·1	1·1	0·9, 1·4	1·1	0·9, 1·3
Mother characteristics						
Education						
Secondary or above	1·0		1·0		1·0	
Primary	1·2	0·9, 1·6	1·1	0·8, 1·6	1·3	0·9, 1·8
Unschooled	1·3	1·0, 1·6	1·2	0·9, 1·6	1·4	1·0, 1·9
BMI (kg/m²)						
Normal (18·5–24·9)	1·0		1·0		1·0	
Thin (<18·5)	1·3	1·0, 1·6	2·0	1·5, 2·6	1·9	1·5, 2·3
Overweight (≥25)	0·8	0·7, 1·0	0·9	0·7, 1·1	0·7	0·5, 0·8

## Discussion

This study analysed the factors associated with stunting, wasting and underweight in children under 5 years of age in Guinea between 2005 and 2018 to identify areas for improvement to enable children to grow up in good health. During the period 2005–2018, Guinea has experienced several events that have negatively affected the nutritional status of households and therefore of children. First, the country has experienced a destabilisation of the political and socio-economic situation since 2008, which has led to a sharp deterioration in the food situation of households^(16)^. Second, the Ebola virus disease epidemic has severely affected the nutritional status of children in two ways. Firstly, many have lost one or both parents and have been orphaned. Second, the epidemic has had a catastrophic impact on already fragile health systems. In addition, the decline in income, the disruption of trade, commercial flights and harvesting activities and also the quarantine measures have increased the food insecurity of the majority of families^(17)^. In response to these events, several interventions have been implemented by the government, institutions and non-governmental organisations operating in the nutrition and food security sector. For example, the strategic collaboration between the World Food Programme and UNICEF was very dynamic in the period before the Ebola crisis. In addition, representatives of both agencies have been very active and have helped to focus attention on nutrition at the highest level of government. Guinea’s membership of Scaling Up Nutrition and Renewed Efforts Against Child Hunger and undernutrition is testimony to their efforts^(18)^.

Analysis of the dataset showed that rural residence, low household socio-economic status, age and sex were the factors associated with stunting in children. Low maternal weight was the only factor associated with wasting. Household wealth quintile, age, occurrence of diarrhoea and fever in the last 2 weeks preceding the surveys, and low maternal weight were significantly associated with underweight children.

This study showed a higher likelihood to stunting and underweight in children from the poorest and poor categories. The reasons for poor nutritional status among poor communities could be their standard of living, unfavourable environmental conditions, low purchasing power and low use of health care services^(5,19)^. In addition, these families are often not in a position to spend more money to provide their children with a healthy and adequate diet, which could make them vulnerable to all these forms of malnutrition.

This study showed an age-dependent divergent trend between stunting and wasting. The likelihood of children to stunting increased with age, while the likelihood to wasting decreased. A study has reported that periods of wasting and weight fluctuations in childhood increase the risk of stunting in later life^(20,21)^. Other studies also indicate that when a child is treated for severe wasting, growth in height slows down until weight is restored, indicating that the body adapts itself to poor weight gain by slowing growth^(22,23)^. This information highlights the role that prevention and treatment of wasting can play in promoting the physical growth of children^(24)^.

Boys were more likely to be stunted than girls. This finding is confirmed by a systematic review of stunting, wasting and underweight in sub-Saharan Africa^(3)^ and a meta-analysis of sixteen DHS in sub-Saharan Africa^(25)^ but contradicted by a study in Kenya which reported that female were more likely to be stunted^(26)^. Boys’ vulnerability to stunting may be explained by less attention than girls^(27)^, despite the fact that boys need more calories for growth and development^(28)^.

Similarly, low-weight maternal increased the risk of stunting, wasting and underweight among children. Poor maternal nutrition leads to poor intra-uterine growth and low birth weight. Undernourished mothers cannot breastfeed their children properly, which exposes their children to poor nutrition^(29)^. Therefore, improving maternal health is a prerequisite for reducing malnutrition in children^(29)^. The association between stunting, wasting, underweight and maternal characteristics (education and maternal BMI) has also been observed in other studies^(30–32)^.

In view of these results, there is still much to be done to improve the nutritional status of children. For this to happen, policymakers must work more to fight poverty in all its forms. Awareness-raising activities on the nutritional practices of parents and children must be intensified. In addition, actions on the promotion of exclusive breast-feeding and the initiation and support of mothers in the diversification of their children’s diet must continue to take place.

The study has some limitations, mainly the lack of the weight and height data for most of the children surveys, which is due to DHS being household surveys; data are collected for all children under 5 years of age living in the selected households, but weight and height measurements are taken for children present on the day of the survey. In addition, the proportion of missing data was similar in all three surveys. This must be taken into account in future surveys in order to reduce bias in the estimation of prevalence. Another point is that it was not possible to establish a causal link as the data used herein were from a prevalence survey; cohort studies would be more adapted for this; however, the present study could enable policymakers to take effective and sustainable action to reduce the occurrence and consequences of malnutrition in children. It also raises the issue of inequality allowing authorities to set priorities and target interventions. Furthermore, the data analysed are from national surveys, and therefore, the results can be extrapolated to the entire Guinean population.

## Conclusion

The nutritional status of children under 5 years of age remains a concern in Guinea. Significant associations were observed between stunting, wasting and underweight and certain characteristics of the child, the mother and the household that can be targeted by governmental policies to improve the nutritional status of children and mothers.
